# Parent–child attachment and mental health in young adolescents: a moderated mediation analysis

**DOI:** 10.3389/fpsyg.2023.1298485

**Published:** 2023-12-22

**Authors:** Rong Tan, Yizhi Yang, Tao Huang, Xuanxuan Lin, Hua Gao

**Affiliations:** ^1^School of Psychology, Fujian Normal University, Fuzhou, China; ^2^Yiyang Normal College, Yiyang, China; ^3^Department of Preschool Education, Jiangmen Preschool Education College, Jiangmen, China; ^4^Institute of Education Sciences, Huazhong University of Science and Technology, Wuhan, China

**Keywords:** adolescents, parent–child attachment, psychological quality, coping styles, mental health, regulatory mediators

## Abstract

**Introduction:**

The parent–child attachment has a significant impact on adolescents’ mental health. However, the influence of psychological quality and coping styles on this connection remains unknown. This study examined the relationship between parent–child attachment and adolescent mental health, by exploring the mediating role of psychological quality and the moderating role of coping styles.

**Methods:**

A total of 633 young adolescents participated in this study after signing informed consent. They anonymously completed questionnaires including the Parent and Peer Attachment Scale (Parent Attachment Section), the Coping Styles Inventory for Middle School Students, the Brief Version of the Psychological Quality Inventory for Middle School Students, and the Chinese Middle School Students’Psychological Quality Inventory. After controlling for gender, grade, left-behind category, only-child status, and family structure.

**Results:**

The moderated mediation model yielded the following findings: (a) parent–child attachment significantly and positively predicted adolescents’mental health; (b) psychological quality partially mediated the relationship between parent–child attachment and adolescents’ mental health; (c) the association between psychological quality and mental health was moderated by task-focused coping.

**Discussion:**

This moderation effect was more substantial for students with low task-focused coping behaviors, which aligns with the “exclusionary hypothesis” model. Therefore, our results indicate that parent–child attachment indirectly impacts mental health, influenced by internal and external factors. These findings carry significant implications for safeguarding and promoting adolescents’ mental well-being.

## Introduction

Mental health refers to a state of internal and external equilibrium and harmonization, enabling individuals to effectively manage positive or negative emotions and adapt to society (Galderisi et al., 2015). Research on mental health indicates a concerning increase in the prevalence of mental illness among China’s youth population in recent years (Yu et al., 2022; Zhang et al., 2022). A recent systematic epidemiological survey of children and adolescents in China revealed that the prevalence of psychiatric disorders had reached an all-time high compared to previous data reported by the Chinese media. The prevalence of these diverse disorders, including attention-deficit disorder (10.2%), anxiety disorders (4.7%), convulsive disorders (2.5%), and major depressive disorders (3.0%), should be a cause for concern for the community (Li et al., 2022).This rise highlights significant challenges faced by youth in maintaining good mental health, In particular, Internalized psychology factors such as psychological distress, self-acceptance (Hsieh et al., 2014) and externalized behavior factor (Li et al., 2014; Huang et al., 2016; Ding et al., 2018) (e.g., Internet addiction, sexual abuse experience and alcohol abuse) will significantly affect teenagers to keep good mental health, which can adversely impact their educational achievement (Dalsgaard et al., 2020), personal development, and even increase the risk of suicide (Coffman and Swank, 2021). Therefore, it is crucial to prioritize the mental well-being of adolescents. In this regard, Some researchers in the field of positive psychology emphasizing the importance of nurturing positive qualities in individuals alongside addressing psychological issues (Keyes and Lopez, 2009; Keyes et al., 2011). This perspective highlights the need for researchers to not only focus on psychological problems but also consider the positive aspects of individuals. Therefore, researchers should concentrate on preventing and intervening in adolescent mental health, but also prioritize maintaining and improving their overall mental well-being.

Parent–child Attachment refers to the emotional bond that parents develop with their children during the process of raising them (Bowlby, 1969). This bond profoundly influences the individual’s physical and mental well-being (Ranson and Urichuk, 2008; Risi et al., 2021), and its impact remains pivotal during adolescence, despite the increasing diversification of attachment forms, including the growing importance of peer attachment (Gorrese and Ruggieri, 2012). Adolescence is a stage of psychosocial risk related to with an increased risk of poor academic adjustment (Veiga et al., 2023), drug use (Liu et al., 2022) or behavioral problems (Baumrind, 1991), in part due to the influence of the family decreases while that of peers increases (Steinberg and Morris, 2001; Reyes et al., 2023). However, it appears that attachment to peers tends to be based on family experiences (Lamborn et al., 1991; Alcaide et al., 2023). Those adolescents with parents who maintain a trusting relationship with their parents, based on responsive parenting, tend to reproduce positive models with their peers and show less involvement in risky behaviors (Darling and Steinberg, 1993; Martinez-Escudero et al., 2023). In contrast, attachment to peers based on deviant standards tends to appear in those adolescents raised in homes with uninvolved parents (Steinberg, 2001; Villarejo et al., 2023). Research indicates that adolescents with low-quality parent–child attachment are more likely to seek attachment to their peers. However, it has been observed that high-quality peer attachment does not compensate for the lack of mental health benefits for those with low-quality parent–child attachment, and peer attachment plays more of a moderating role in the effects of parent–child attachment on adolescent mental health (Zhang and Deng, 2022; Chen et al., 2023). This indicates that although parent–child attachment may not be the exclusive form of attachment for adolescents, it continues to be a critical factor in their mental well-being. Enhancing the parent–child relationship can therefore serve as a means to prevent adolescent health risk behaviors (Ackard et al., 2006). Empirical studies have demonstrated that low-quality parent–child attachment is associated with internalized psychological problems such as loneliness, depression (Yıldız, 2016), and emotional anorexia nervosa (Laporta-Herrero et al., 2021). adolescents with high-quality parent–child attachment tend to exhibit higher self-efficacy (Chen et al., 2019) and better mental health (Liu et al., 2019; Spruit et al., 2020). Hypothesis 1: Parent–child attachment is a significant positive predictor of adolescent mental health.

However, exploring the direct relationship between parent–child attachment and adolescent mental health alone does not address the underlying mechanisms of how parent–child attachment operates. Therefore, it is necessary to further investigate the mediating effect between parent–child attachment and mental health. Psychological quality refers to an individual’s stable and fundamental psychological characteristics that are shaped by external stimuli and closely linked to their adaptation, development, and creative behaviors (Zhang et al., 2011a). This concept is considered a positive psychological attribute and holds significant recognition and value in the study of Chinese adolescents’ mental health. It is often described as an indirect factor in the relationship between various mental health influences (such as school climate and family functioning) and the mental well-being of students within Chinese culture (Nie et al., 2020; Zhang and Wang, 2020). It plays a crucial role in influencing mental health factors directly or indirectly (Lucas et al., 2008; Suldo and Shaffer, 2008; Keyes and Lopez, 2009). Research indicates that psychological quality significantly and negatively predicts psychological symptoms such as stress or depression (Taylor et al., 2003; Roisman et al., 2012), while also predicting positive mental health indicators like positive emotions (Koh et al., 2008). Generally, individuals with high levels of psychological attributes tend to demonstrate higher levels of mental health compared to those with low psychological attributes (Wang and Zhang, 2012a,b; Wang et al., 2015). These findings suggest that psychological quality can significantly impact mental health, potentially exerting a protective effect.

Bowlby posits that parent–child attachment directly impacts the development and integrity of an individual’s personality (Bowlby, 1969, 1982). Adolescents with high-quality parent–child attachment tend to exhibit elevated levels of self-esteem, self-efficacy, and emotional regulation (Wilkinson, 2004; Li et al., 2015; Chen et al., 2019). These psychological traits are considered essential components of an individual’s psychological quality, implying that psychological quality plays a pivotal role in personality development (Wang et al., 2015). Furthermore, it has been observed that psychological quality mediates the relationship between parent–child attachment and life satisfaction as well as depression (Chen et al., 2016, 2017). Since life satisfaction and depression are significant constructs linked to mental health, this suggests that psychological quality may serve as a “bridge” between parent–child attachment and mental health; in other words, parent–child attachment may influence adolescent mental health by shaping psychological quality. To summarize, Hypothesis 2 is proposed: Psychological quality mediates the relationship between parent–child attachment and adolescent mental health.

The resilience model of child development posits that adverse circumstances, such as low-quality parent–child attachment, do not necessarily lead to poor child development (e.g., low mental health). The ultimate developmental outcome largely depends on the availability of protective factors that individuals possess to cope with adversity (Luthar et al., 2000). While investigating the mediating role of psychological attributes, it is crucial to explore the role of protective factors further. Coping strategies encompass the cognitive and behavioral efforts individuals employ to mitigate the negative effects of stress. They can be categorized into task-focused coping, which involves positive strategies for problem-solving, positive rationalization, and seeking social support, and emotion-focused coping, which tends to employ negative strategies such as endurance, avoidance, emotional venting, and denial (Worsley et al., 2019). The former is good at using positive strategies(problem solving, positive rationalization, and seeking social support) to copy and adapt to environmental changes, while the latter tends to cope with environmental changes with negative strategies (endurance, avoidance, venting emotions, and denial of fantasies) (Chen et al., 2000). Numerous studies have emphasized the prominent protective effect of adaptive coping styles on mental health (Compas et al., 2014, 2017). Similar to psychological quality, coping styles are regarded as protective factors that aid individuals in navigating unfavorable situation (Imran et al., 2021). Furthermore, according to the “protective factor-protective factor model” of human development (Bronfenbrenner, 1979, 1986), dual protective factors interact with the outcome variable, indicating that one protective factor moderates the relationship between the other protective factor and the outcome variable (Fuentes et al., 2020). Exploring the moderating role of coping styles in the relationship between psychological quality and mental health is therefore crucial for understanding “when psychological quality is most effective”.

**Figure 1:**
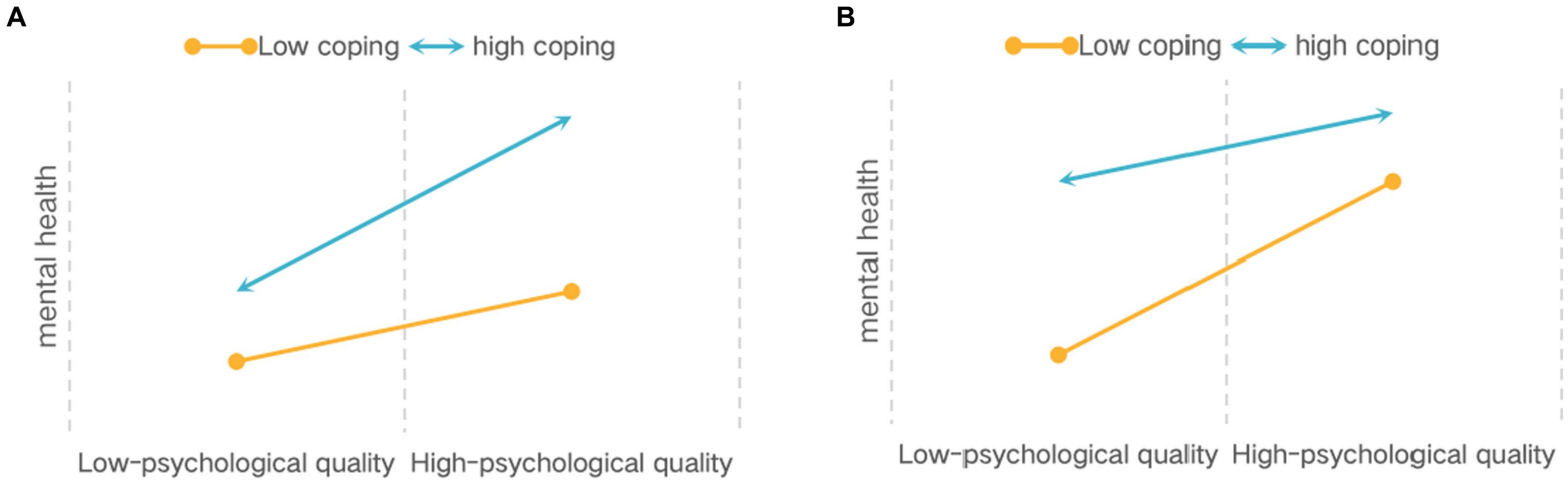
Two hypotheses of the “protective factor model”. **(A)** “promotion hypothesis”. **(B)** “exclusion hypothesis”.

The moderating role of coping styles can be understood through two possible models: the facilitation hypothesis and the exclusion hypothesis. In the “promotion hypothesis” model, coping styles amplify the impact of psychological quality on mental health. In other words, the protective effect of psychological quality is more pronounced when individuals exhibit high levels of problem-focused or emotion-focused coping compared to low levels (refer to Figure 1A; Jessor et al., 1995). In the exclusion hypothesis model, coping styles weaken the effects of high-psychological quality on mental health. Here, the protective effect of psychological quality is more likely to be observed in individuals with low-psychological quality rather than high psychological quality converse to facilitation hypothesis (refer to Figure 1B; Jessor et al., 1995). Due to the limited number of studies exploring the interaction between coping styles and psychological quality, specific hypotheses about the regulatory mechanisms are challenging to formulate. Instead, Hypothesis 3 is proposed: Coping styles moderate the latter part of the mediating pathway through which parent–child attachment influences psychological well-being via psychological quality.

**Figure 2:**
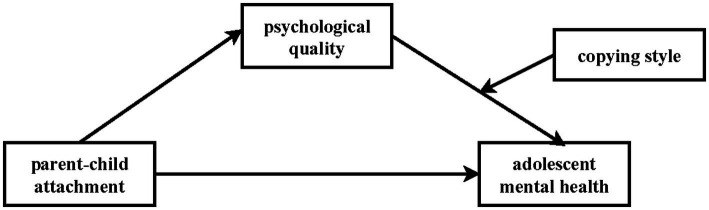
Hypothetical model.

In summary, this study adopts the theoretical framework of attachment theory, the model of the relationship between psychological quality and mental health, and the protective factor-protective factor model of human development to establish a moderated mediation model (refer to Figure 2). This model aims to elucidate the influence of parent–child attachment on adolescent mental health. By exploring this mediation model (refer to Figure 2), the study seeks to uncover the underlying mechanism through which parent–child attachment affects the mental health of adolescents. The findings are expected to provide empirical evidence for interventions and protective measures targeted at promoting the mental health of adolescents.

## Materials and method

### Participants

A cluster sampling method was utilized to select adolescents from junior to senior grades in Hunan, Fujian, and Jilin provinces (Includes 3 schools and 18 classes, with 6 classes selected from each school). The questionnaires were distributed through an online platform called Questionnaire Star, and participants were provided with compensation (Each participant will receive 5 RMB after completion)for completing the questionnaires. All participants signed informed consent, and if participants are less than lower 18 years old we will get their parents’ informed consent meanwhile. A total of 697 questionnaires were collected, of which 633 were deemed valid, resulting in an effective response rate of 90.8%. Among the participants, 254 (40.1%) were male, and 379 (59.9%) were female. In terms of grade level, 133 (21.0%) were in the first grade, 106 (16.7%) in the second grade, 111 (17.5%) in the third grade of junior high school, 83 (13.1%) in the first grade of senior high school, 115 (18.2%) in the second grade, and 85 (13.4%) in the third grade of senior high school. Regarding residential areas, 333 (52.7%) resided in towns and cities, while 300 (47.3%) lived in rural areas. There were 175 (27.8%) only children and 457 (72.2%) with siblings. In terms of parent’s marital status, 562 (88.8%) had parents in normal marital status, 29 (4.6%) had single parents, 30 (4.7%) had divorced parents, and 12 (1.9%) had parents in other family arrangements.

### Measure

#### Parent–child attachment

Parent–child Attachment was assessed by The Inventory of Parent and Peer Attachment (IPPA-R) Chinese version scale (Zhang et al., 2011b). The scale consists of two subscales: father-child attachment (e.g., I think I have a good father.) and mother–child attachment (e.g., I want someone else to be my mother.), and three sub-dimensions: trust, communication, and alienation. Participants rated their responses on a Likert 5-point scale ranging from 1 (never like this) to 5 (always like this). The total score was calculated by summing the scores of the two subscales, with higher scores indicating better quality of parent–child attachment. The Cronbach’s alpha coefficients for mother–child attachment were 0.88 (trust), 0.87 (communication), and 0.79 (detachment), and for father-child attachment were 0.89 (trust), 0.89 (communication), and 0.79 (detachment).

#### Adolescent coping styles

The Adolescent Coping Styles Questionnaire (ACSQ) scale, developed by Chen et al. (2000), is a self-report measure consisting of 36 items(e.g., Seek advice from experienced or similarly experienced people). It assesses two coping dimensions: task-focused coping and emotion-focused coping. Task-focused coping comprises three factors: problem-solving, seeking social support, and positive rationalization. Emotion-focused coping comprises four factors: endurance, avoidance, venting emotions, and fantasy denial. Respondents rate their frequency of using coping strategies on a 4-point scale ranging from 1 (not used) to 4 (often used). Subscale scores are computed by summing the scores obtained from the corresponding factor items. The Cronbach’s alpha coefficient for task-focused coping was 0.90, and for emotion-focused coping, it was 0.87.

#### Psychological quality

The Psychological Quality Scale for Middle School Students (simplified version) scale was revised by Hu et al. (2017). The scale consists of 24 items (e.g., I often base my approach on the learning task), encompassing three dimensions: cognitive traits, personality qualities, and adaptive ability. All items are positively scored on a Likert 5-point scale, ranging from “very non-compliant” to “very compliant,” with higher scores indicating higher levels of psychological quality. The questionnaire demonstrated high internal consistency, with a Cronbach’s alpha coefficient of 0.95.

#### Mental health

The Mental Health Inventory for Chinese Middle School Students (MMHI-60) scale, developed by Wang et al. (1997), is considered an ideal measure to assess the mental health status of adolescents. This scale comprises 60 items (e.g., Doing homework must be double-checked), which are grouped into 10 factors: obsessive-compulsive symptoms, paranoia, hostility, interpersonal tension and sensitivity, depression, anxiety, learning stress, maladjustment, emotional imbalance, and psychological imbalance. Each item is rated on a 5-point scale. Subscale scores are calculated as the mean scores of the corresponding items, while the total score represents the mean score of all items. It’s worth noting that, higher scores on the scale indicate lower levels of mental health. The Cronbach’s alpha coefficient for the mental health scale was reported to be 0.98, indicating high internal consistency.

#### Procedure

Middle school students from various schools voluntarily agreed to take part in this survey. Prior to completing the questionnaire, the researcher provided an explanation regarding the survey’s importance, emphasized its anonymity, and assured participants that there were no right or wrong answers. Meanwhile, the parents of any participant will sign informed consent. Participants were encouraged to answer honestly and informed of their right to withdraw from the survey at any time. The completion time for all the questionnaires averaged around 30 min. To ensure data quality, questionnaires that were completed in less than 340 s were excluded, based on the criterion that each question should take at least 2 s to answer (Huang et al., 2012).

### Statistics

The data were analyzed using SPSS 25.0 software and SPSS PROCESS macro program. The analysis involved several steps. First, descriptive statistics were computed, and a Pearson correlation analysis was conducted. Second, after standardizing all the data, regression analyzes were performed following the recommended process by previously recommended method (Wen and Ye, 2014). Specifically, (a) a regression analysis of mental health on Parent–child Attachment was conducted, (b) a regression analysis of psychological quality on Parent–child Attachment was conducted, and (c) a regression analysis of both and was performed. Third, using 5,000 bootstrap samples (Hayes and Scharkow, 2013), a process macro (Model 4) was utilized to test whether coping style moderates the mediating process. A significant effect is indicated when the confidence interval does not include zero.

## Results

Because this study collected data through self-reporting methods, it was possible that there could be an issue with common method variance (CMV). To reduce this possible deviation, according to the suggestion by The Harman one-way test was used to test the research questionnaire for common method bias (Tehseen et al., 2017), which found that there were 30 common factors with eigenvalues greater than 1, and the rate of explanation for analyzing the first common factor was 23.09%, which was much smaller than the critical value of 40%, indicating that there was no serious common method bias in the data of this study.

### Descriptive statistics and correlation analysis

Correlation analyzes (see Table 1) showed that father/mother–child attachment and its dimensions, parent–child attachment, psychological quality and its dimensions, and task-focused coping were significantly and positively correlated, and MMHI-60 scores was significantly and negatively correlated with all of the above variables and dimensions, while it was significantly and positively correlated with emotional-centered coping (*p* < 0.01).

**Table 1:** Descriptive statistics and correlation analysis.

Variant	*M*	SD	1	2	3	4	5	6	7
1. Gender	/	/	1						
2. Grades	/	/	−0.02	1					
3. Parent–child attachment	82.96	24.48	0.11^**^	−0.07	1				
4. Psychological quality	86.19	16.83	0.14^**^	−0.20^**^	0.49^**^	1			
5. Task-focused Coping	56.56	9.67	0.07	−0.02	0.51^**^	0.73^**^	1		
6. Emotion-focused Coping	40.82	9.05	0.05	0.10^*^	0.05	0.05	0.28^**^	1	
7. MMHI-60	2.21	0.81	−0.10^*^	0.12^**^	−0.33^**^	−0.38^**^	−0.24^**^	0.40^**^	1

*n* = 633; gender is a dummy variable, male = 1, female = 0, mean indicates proportion of males; MMHI-60 = Total score of The Mental Health Inventory for Chinese Middle school Students scale; ^*^p < 0.05,^**^p < 0.01,^***^p < 0.001.

### The mediating role of psychological quality role

The present study aimed to examine the mediating role of psychological quality in the relationship between father/mother/parent–child attachment and MMHI-60 scores, as there was a significant correlation between these variables, thereby fulfilling the requirements for conducting a mediation analysis. The recommended process, as outlined by previous research methods (Wen and Ye, 2014), was followed to test the mediation effect. Firstly, a regression analysis was performed with the dependent variable MMHI-60 and the independent variable father/mother/parent–child attachment. Secondly, a regression analysis was conducted with the mediator variable psychological quality and the independent variable father/mother/parent–child attachment. Lastly, a regression analysis was carried out with the dependent variable MMHI-60 and the independent variable father/mother/parent–child attachment, along with the mediator variable psychological quality.

The results are shown in Table 2. In Model 1, the effect of parent–child attachment on MMHI-60 was significant (*β* = −0.27, *t* = − 6.01, *p* < 0.001), suggesting that the higher quality of parent–child attachment indicated the lower MMHI-60 scores (higher mental health); in Model 2, the effect of parent–child attachment on psychological quality was significant (*β* = 0.15, *t* = 4.85, *p* < 0.001), suggesting that the higher parent–child attachment indicated better individual’s psychological quality; in Model 3, the effect of parent–child attachment on psychological health was still significant (*β* = −0.21, *t* = −4.83, *p* < 0.001), indicating that psychological quality partially mediates the relationship between parent–child attachment and mental health. The same method was tested as above, with a sample of 5,000, and the upper and lower limits of the Bootstrap 95% confidence interval for this mediating effect did not contain 0 (see Table 3).

**Table 2:** Tests of the mediating effect of psychological quality on the relationship between parent–child attachment and psychological well-being.

variant	Model 1: Mental Health		Model 2: Psychological quality		Model 3: Mental Health	
	*β*	*t*	*β*	*t*	*β*	*t*
Parent–child attachment	−0.27	−6.01^***^	0.15	4.85^***^	−0.21	−4.83^***^
Psychological quality (in ideological education)					−0.39	−7.28^***^
*R^2^*	0.11		0.54		0.17	
*F*	37.92^***^		377.88^***^		45.05 ^***^	

**Table 3:** Analysis of the mediating effect of psychological quality in the impact of mother–child attachment on mental health.

Type of effect	Efficiency value	Boot Standard Error	Lower 95% CI	95% CI ceiling
Aggregate effect	−0.26	0.04	−0.35	−0.20
Direct effect	−0.22	0.04	−0.22	−0.07
Mediating effects of psychological quality	−0.04	0.02	−0.18	−0.09

### The moderating effect of coping styles

According to Wen and Ye (2014) recommended process for testing, a mediating effect of moderation exists if the following four conditions are met (the moderator variable moderates the second half of the mediation path): (1) the effect of father-child attachment on mental health is significant in Model 1; (2) the effect of father-child attachment on psychological quality is significant in Model 2; (3) the effect of psychological quality on mental health is significant in Model 3; and (4) the effect of problem or emotion-centeredness in Model 4 The interaction term between coping and psychological quality had a significant effect on mental health.

The results are shown in Table 4. Model 1: Parent–child attachment significantly and negatively predicted MMHI-60 (*β* = −0.27, *t* = −6.01, *p* < 0.001); Model 2: Parent–child attachment significantly and positively predicted psychological quality (*β* = 0.15, *t* = 4.85, *p* < 0.001); Model 3: Psychological quality significantly and negatively predicted MMHI-60 (*β* = −0.39, *t* = −7.28, *p* < 0.001), the above suggests that psychological quality partially mediates in parent–child attachment and mental health, and the Bootstrap 95% confidence interval for the mediating effect is [−0.18,-0.09]. Model 4: The interaction term between task-focused coping and psychological quality significantly and positively predicted mental health (*β* = 0.10, *t* = 3.10, *p* < 0.01), i.e., task-focused coping positively moderated the effect of psychological quality on mental health (see Figure 2).

**Table 4:** Relationship between parent–child attachment and mental health: mediated model test with moderation.

predictor variable	Model 1: Mental health		Model 2: Psychological quality		Model 3: Mental health		Model 4: Mental health	
	β	t	β	t	β	t	β	t
Parent–child attachment	−0.27	−6.01***	0.15	4.85***	−0.21	−4.83***	−0.22	−5.15***
Psychological quality (in ideological education)					−0.39	−7.28***	−0.39	−7.35***
Task-focused Coping Psychological quality							0.10	3.10**
*R*^2^	0.11		0.54		0.17		0.18	
*F*	37.92***		377.88***		45.05***		34.67***	

Model 4: There is no significant relationship between the interaction term of emotion-focused coping and psychological quality (*β* = −0.026, *t* = −0.891, *p* > 0.05), indicating that the effect of psychological quality on mental health is not moderated by emotion-focused coping (Table 5).

**Table 5:** Moderating effects of task-focused coping on psychological fitness and mental health under parent–child attachment.

Moderator variable	Efficiency value	Boot standard error	Lower 95% CI	95% CI ceiling
PC				
Z ≤ -1.00	−0.24	0.04	−0.31	−0.17
1.00 < Z < 1.00	−0.19	0.03	−0.26	−0.13
Z ≥ 1.00	−0.14	0.04	−0.22	−0.07

The effect of task-focused coping on the moderating pattern between psychological quality and mental health was further revealed through simple slope analyzes, with interaction effects plotted using the Excel macro file Mod Figure. Simple slope tests showed that when task-focused coping scores were low (-1SD), the predictive effect of psychological quality on MMHI-60 was significant (*β* = −0.52, *t* = −8.35, *p* < 0.001), and that for every 1 standard deviation increase in psychological quality, MMHI-60 decreased by 0.52 standard deviations; when task-focused coping scores were high (+1SD), the predictive effect of psychological quality on MMHI-60 remained significant as a predictor of mental health (*β* = −0.36, *t* = −5.93, *p* < 0.001), but at this point, for every 1 standard deviation increase in psychological quality, MMHI-60 decreased by 0.36 standard deviations, a decrease in the magnitude of the increase relative to when task-focused coping scores were high. This suggests that the effect of psychological quality on MMHI-60 score decreases with increasing task-focused coping tendencies, i.e., the indirect effect of father/mother sub-attachment on MMHI-60 through psychological quality diminishes with increasing task-focused coping tendencies. Therefore, this interaction pattern belongs to the exclusion pattern in the Protective Factor-Protective Factor Model.

## Discussion

The present study aims to construct a moderated mediation model to clarify how parent–child attachment predicts adolescent mental health through the mediating role of psychological quality. Additionally, the study seeks to determine the specific conditions under which psychological quality, particularly low levels of task-focused coping, exerts a stronger influence on adolescent mental health.

### The relationship between parent–child attachment and mental health

The study’s results indicated a significant and positive relationship between parent–child attachment and adolescents’ mental health, which aligns with previous research findings (Yan et al., 2018). High-quality parent–child attachment was found to foster positive mental health outcomes in adolescents, while low-quality attachment was associated with increased internalizing psychological problems and externalizing behavioral issues among adolescents (Yıldız, 2016; Chen et al., 2019; Laporta-Herrero et al., 2021). Secure attachment is a fundamental component of individuals’ psychological security. It influences how individuals respond to others and their emotional experiences in various situations. When parents consistently provide timely and positive responses to their children, it facilitates the establishment of a high-quality parent–child attachment. In this nurturing environment, children feel secure and are encouraged to explore the external world, fostering their socialization, social adaptation, and overall healthy psychological development (Yıldız, 2016). Additionally, a loving environment that promotes security enhances the child’s inclination to explore and supports their future socialization and adaptation.

Although mental health may be affected especially in adolescence a time of increased psychosocial vulnerability (Steinberg, 2001; Reyes et al., 2023), as in childhood the family can be beneficial even as its influence increases (Lamborn et al., 1991; Villarejo et al., 2023) Healthy relationships are based on trust and a family climate of dialog and affection could provide security and self-confidence, especially when there is some developmental maladjustment, as this study seems to indicate in line with other work on the family (Darling and Steinberg, 1993; Martinez-Escudero et al., 2023).

As for family structure, a number of studies support that parental marital status affects parent–child relationships to a certain extent in Chinese culture, for example, adolescents with non-integrated structures are more prominent in depression and deviant behaviors (Yang and Jiang, 2023). Not only that, The family environment has also been found to be deeply linked to juvenile criminal behavior (Deng and Roosa, 2007). Students with non-intact family structures in this study had lower quality of parent–child attachment compared to students with intact family structures, and in agreement with others’ research, these individuals with non-intact family structures are more prone to crisis of trust and further rigidity in parent–child relationships (Ying et al., 2015). On The other hand, students with different family structures showed differences in their emotion-focused coping styles, with students with intact family structures showing fewer direct emotional outbursts, fantasy denial, and other emotional behaviors than students with incomplete family structures. This has also been shown in several studies (Wills et al., 2014).

Furthermore, in the present study, there was no significant difference between the effects of paternal and maternal attachment on adolescents’ mental health, which may be due to the fact that the focus of this paper was on exploring a model of the relationship between overall parent–child attachment on mental health. Previous research has shown that paternal attachment has a stronger effect on a single mental health factor, adolescent depressive symptoms, than maternal attachment (Pan et al., 2016). Although it is common in clinical practice to see the different effects of paternal and maternal attachment on adolescent psychology and behavior, some scholars still give a different point of view, preferring to define the roles played by both in the developmental history of adolescents as complementary to each other, rather than contrasting and delineating between paternal and maternal attachment (Wong et al., 2020). Conversely, adolescents with low-quality parent–child attachment, lacking sufficient security, may experience negative outcomes, leading to a decline in mental health and an increase in internalized or externalized psychological and behavioral problems. Thus, high-quality parent–child attachments offer numerous advantages that cannot be matched by low-quality attachments. Therefore, it is crucial for parents to prioritize the cultivation of high-quality attachment relationships with their children. Building trust and providing positive support will ensure that children develop a secure working model to navigate future academic and personal challenges with enhanced safety and confidence.

### Mediating effect of psychological quality

The present study found that psychological quality plays a partial mediating role in the relationship between parent–child attachment and psychological well-being. The present findings align with existing studies that highlight the importance of high-quality parent–child attachment for adolescents. Adolescents who experience a secure and nurturing parent–child attachment relationship tend to develop a sense of security, which serves as a “safe base” to actively explore new situations and foster personal growth (Bowlby, 1982). Furthermore, the experience of a positive parent–child relationship can contribute to the improvement of the child’s psychological quality (Chen et al., 2019), confirming the fundamental concepts of attachment theory.

Compared to adolescents with low-quality parent–child attachment, those with high-quality attachments demonstrate higher levels of self-esteem, self-efficacy, and emotional regulation (Murphy et al., 2015). This emphasizes the significant influence of high-quality parent–child attachment on enhancing adolescents’ psychological well-being and overall psychological quality. When individuals have their basic sense of security fulfilled, they are more likely to acquire positive qualities that contribute to psychological well-being, facilitating better adjustment to society, education, and life (Wang et al., 2015).

Additionally, the results support the proposed model linking psychological quality and mental health. Adolescents with high-quality parent–child attachments are more likely to develop elevated levels of psychological quality. This, in turn, enables them to effectively manage their emotions and behaviors, leading to enhanced interpersonal relationships (Wang and Zhang, 2012a,b) and psychological well-being (Li et al., 2015; Chen et al., 2019). Conversely, adolescents who experience low-quality parent–child attachment may struggle to satisfy their basic security needs, thus manifesting low self-esteem, low self-efficacy, low self-control, and poor emotional regulation (Murphy et al., 2015; Morris et al., 2017). Consequently, they are more susceptible to lower levels of psychological quality and subsequent psychological issues (Taylor et al., 2003; Koh et al., 2008; Roisman et al., 2012). In conclusion, the findings emphasize how high-quality parent–child attachment plays a pivotal role in shaping adolescents’ psychological quality, mental health, and overall well-being.

### Moderating effect of task-focused coping

The results of the study demonstrated that task-focused coping moderated the second half of the mediation pathway “parent–child attachment → psychological quality → mental health.” This moderation pattern followed the “exclusionary hypothesis” model, partially confirming previous study findings (Guo et al., 2018; Imran et al., 2020, 2021). These results are consistent with the “protective factor-protective factor model” of human development (Fuentes et al., 2020). Specifically, for adolescents with low task-focused coping tendencies, the impact of psychological quality on mental health was stronger. Conversely, for adolescents with high task-focused coping tendencies, the impact of psychological quality on mental health was weaker. Similar findings have been reported in previous studies (Besharat et al., 2008).

These findings suggest that task-focused coping strategies can positively moderate the negative effects of stressors on mental health outcomes (in this case, academic burnout). Adolescents who employ task-focused coping tend to face problems directly, actively seek effective solutions, and engage in cognitive appraisal of themselves, others, and the environment, leading to the development of positive psychological qualities. Furthermore, task-focused coping can compensate for low psychological quality, diminishing its negative impact and safeguarding individuals’ psychological well-being (Imran et al., 2020, 2021).

Moreover, the study found no moderating effect of emotion-focused coping on the relationship between psychological quality and mental health. This upholds a viewpoint supported by previous research (Aldwin and Revenson, 1987; Taylor and Stanton, 2007). However, it suggests the possibility of intermediary factors playing a role in influencing the moderating effect of emotion-focused coping. For instance, emotion-focused coping may indirectly impair students’ mental health through negative thoughts or emotions (Besharat et al., 2008). Thus, the findings highlight the importance of addressing adolescents’ task-focused coping strategies to promote their psychological well-being.

### Educational responses and limitations

This study contributes to the existing body of research by further elucidating the mechanism through which parent–child attachment influences adolescent mental health. The findings hold both theoretical value and practical significance. Theoretically, the results support the principles of attachment theory, the model of psychological quality and mental health, and the “protective factor-protective factor” model of human development. These empirical findings provide additional evidence for the impact of parent–child attachment on adolescent mental health, highlighting the intricate relationships between family factors, coping processes, personal traits, and their influence on psychosocial adaptation.

From a practical standpoint, this study sheds light on the protective mechanisms underlying adolescent mental health. It offers empirical support for the development and implementation of mental health education programs for adolescents, taking into account the specific requirements and challenges of this age group. The knowledge gained from this study can guide interventions and practices aimed at promoting the psychological well-being and adaptive functioning of adolescents in various settings. Overall, this research has implications for enhancing adolescent mental health education in accordance with contemporary needs.

Based on the results of the study, several suggestions can be made for adolescent mental health education. First, foster positive parent–child relationships by emphasizing the importance of cultivating positive parent–child relationships within families. Provide resources and support for parents through activities like parent–child group counseling to improve and enhance the quality of these relationships. Second, address weak family support through identifying students with weak family support and provide targeted interventions and support. School mental health teachers can play a crucial role in offering guidance and resources to these students, aiming to mitigate the detrimental effects of poor parent–child relationships on their mental health. Third, focus on psychological quality in middle school: Recognize that middle school is a critical period when adolescents are more vulnerable to psychological problems. Educators should allocate attention and resources to promote and enhance students’ psychological quality during this stage. Help students explore their positive inner qualities, build resilience, and develop effective coping strategies to resist stress. Fourth, promote task-focused coping strategies: Encourage students to utilize task-focused coping strategies to cope with stress. These may include problem-solving, seeking social support, and adopting positive cognitive perspectives to rationalize challenging situations. Efforts should be made to educate students about the benefits of task-focused coping and provide opportunities for them to develop and practice these skills.

By implementing these suggestions, schools and educators can contribute to the well-being and mental health of adolescents. It is crucial to create a supportive environment that facilitates positive parent–child relationships, promotes psychological quality, and equips students with effective coping skills, ultimately enhancing their overall mental health and resilience.

The present study also has several limitations. First, the cross-sectional questionnaire limits the ability to establish causal relationships between parent–child attachment and mental health. Future research would benefit from employing longitudinal or experimental designs to examine the temporal dynamics and establish clearer causal relationships between these variables. Second, the study found that emotion-focused coping did not moderate the mediator model and only served as a direct predictor of mental health. Exploring potential intermediate variables that may influence the moderating effect of emotion-focused coping could provide a deeper understanding of its role. Future research should consider incorporating additional variables that may influence the moderating relationship. The current study focused solely on exploring protective factors for mental health and did not integrate the examination of risk factors. To gain a more comprehensive understanding of the influencing factors, future studies can explore the risk-protective factor model, which incorporates both the risk and protective factors to provide a more holistic perspective on adolescent mental health.

## Conclusion

The findings of this study have significant implications both theoretically and practically. From a theoretical perspective, this study expands on previous research by highlighting the role of psychological quality as a mediating factor in the relationship between parent–child attachment and mental health. This sheds light on the underlying mechanisms that explain how parent–child attachment influences adolescent well-being. It adds to the existing knowledge by emphasizing the importance of psychological factors in shaping the current and future trajectories of middle school students across various aspects of their lives, including learning and behavior. From a practical perspective, the study’s findings suggest that the development of psychological quality and task-focused coping strategies holds crucial importance for adolescent mental health (Jozan et al., 2021; Ataei et al., 2023). Educators and practitioners can leverage these findings to support the well-being of middle school students. Specifically, it is essential for families to prioritize the cultivation of positive parent–child relationships by fostering trust and effective communication (López Turley et al., 2010; Qu et al., 2015).

Furthermore, focusing on the development of students’ psychological quality can be a key strategy. Educators can inspire students to discover and nurture their intrinsic qualities, enhancing their self-awareness (London et al., 2023) and resilience (Yeager and Dweck, 2012). Additionally, promoting task-focused thinking and encouraging a positive response to academic and life challenges through teaching and learning strategies can contribute to reducing the occurrence of psychological problems (Blount et al., 1991; Williams et al., 2017). As a long-term solution, integrating mental health education as a regular part of the curriculum can be considered (Smith et al., 2006). Providing students with the necessary knowledge, skills, and support structures will empower them to improve and maintain their mental well-being.

In summary, the study’s findings suggest practical approaches to enhancing adolescent mental health by emphasizing positive parent–child relationships, developing psychological quality, and promoting task-focused coping strategies. By implementing these strategies, educators and families can contribute to the overall mental well-being of middle school students.

## Data availability statement

The data analyzed in this study is subject to the following licenses/restrictions: Requests to access these datasets should be directed to RT, 745580311@qq.com.

## Ethics statement

This study was approved by the Ethics Committee of Fujian Normal University. The studies were conducted in accordance with the local legislation and institutional requirements. Written informed consent for participation was not required from the participants or the participants’ legal guardians/next of kin in accordance with the national legislation and institutional requirements.

## Author contributions

RT: Conceptualization, Writing – original draft, Data curation, Formal analysis, Writing – review & editing. YY: Conceptualization, Writing – original draft, Data curation, Formal analysis, Writing – review & editing. TH: Data curation, Methodology, Resources, Writing – review & editing. XL: Data curation, Methodology, Formal analysis, Writing – review & editing. HG: Funding acquisition, Supervision, Writing – review & editing.
